# Interference with SRF expression in skeletal muscles reduces peripheral nerve regeneration in mice

**DOI:** 10.1038/s41598-020-62231-4

**Published:** 2020-03-24

**Authors:** Renate Wanner, Bernd Knöll

**Affiliations:** Institute of Physiological Chemistry, Ulm University, Albert-Einstein-Allee 11, 89081 Ulm, Germany

**Keywords:** Somatic system, Regeneration and repair in the nervous system

## Abstract

Traumatic injury of peripheral nerves typically also damages nerve surrounding tissue including muscles. Hence, molecular and cellular interactions of neighboring damaged tissues might be decisive for successful axonal regeneration of injured nerves. So far, the contribution of muscles and muscle-derived molecules to peripheral nerve regeneration has only poorly been studied. Herein, we conditionally ablated SRF (serum response factor), an important myofiber transcription factor, in skeletal muscles of mice. Subsequently, the impact of this myofiber-restricted SRF deletion on peripheral nerve regeneration, i.e. facial nerve injury was analyzed. Quantification of facial nerve regeneration by retrograde tracer transport, inspection of neuromuscular junctions (NMJs) and recovery of whisker movement revealed reduced axonal regeneration upon muscle specific *Srf* deletion. In contrast, responses in brainstem facial motor neuron cell bodies such as regeneration-associated gene (RAG) induction of *Atf3*, synaptic stripping and neuroinflammation were not overly affected by SRF deficiency. Mechanistically, SRF in myofibers appears to stimulate nerve regeneration through regulation of muscular satellite cell (SC) proliferation. In summary, our data suggest a role of muscle cells and SRF expression within muscles for regeneration of injured peripheral nerves.

## Introduction

Traumatic accidents often injure several organs at the same time. In peripheral nerve injuries of e.g. body extremities, nerves are typically not damaged in isolation but neighboring tissues such as muscles providing the surface for nerve trajectories are likewise injured. Thus, after injury of different tissues and cell types, for instance neurons and myofibers, these cell types might interact reciprocally to drive nerve and muscle regeneration. So far, molecular and cellular nature of such regenerative mechanisms are poorly understood. In consequence, only few reports have identified muscle derived molecules or cell types stimulating peripheral nerve regeneration. For instance, muscle restricted overexpression of a neurotrophic factor mixture (VEGF, BDNF, GDNF, IGF-1) improves recovery after nerve injury^1,2^. Furthermore, muscle stem cells may differentiate into myelinating Schwan cells and thereby enhance nerve regeneration^3^. Muscle restricted gene mutagenesis in mice identified single muscle specific molecules such as YAP (Yes-associated protein) of the hippo pathway and ciliary neurotrophic factor (CNTF) receptor α as potential promoters of nerve regeneration^4,5^. Notably, deletion of the muscle spindle resident transcription factor Egr3 impairs both regeneration of axons in the peripheral^6^ and central nervous system^7^. Hence, muscle spindles emerge as important muscular structure in axon regeneration.

A further key transcription factor regulating several aspects of skeletal muscle physiology and pathology is the serum response factor (SRF)^8–11^. As shown by conditional murine *Srf* inactivation restricted to skeletal muscles, SRF deficient myofibers show premature aging, enhanced muscle fibrosis and myofiber specific atrophy resulting in reduced muscle function^12,14–17^. After increased load, SRF function is required for overload induced myofiber hypertrophy^16^. Following such myofiber hypertrophy, SRF ablation in myofibers impinges also on proliferation of Pax7 positive satellite cells (SCs), the muscle resident stem cell population^13,16^. This involved a paracrine mechanism by which SRF in myofibers controls transcriptional regulation and thereby protein secretion of interleukin-6 (Il-6) and cyclooxygenase-2 (Cox2)/interleukin-4 (Il-4) controlling SC proliferation^16,17^. Further SRF target genes relevant to muscle function are genes encoding actin isoforms (skeletal actin, *Acta1*; α-cardiac actin, *Actc1*; β-actin, *Actb*; γ-actin, *Actg* and smooth muscle actin, *Acta2*) or actin binding proteins such as calponin and filamins^9,18^. So far it has not been analyzed whether SRF in muscles stimulates peripheral nerve regeneration. However, muscle restricted SRF ablation augments muscle atrophy after sciatic nerve denervation^19^.

**Figure 1:**
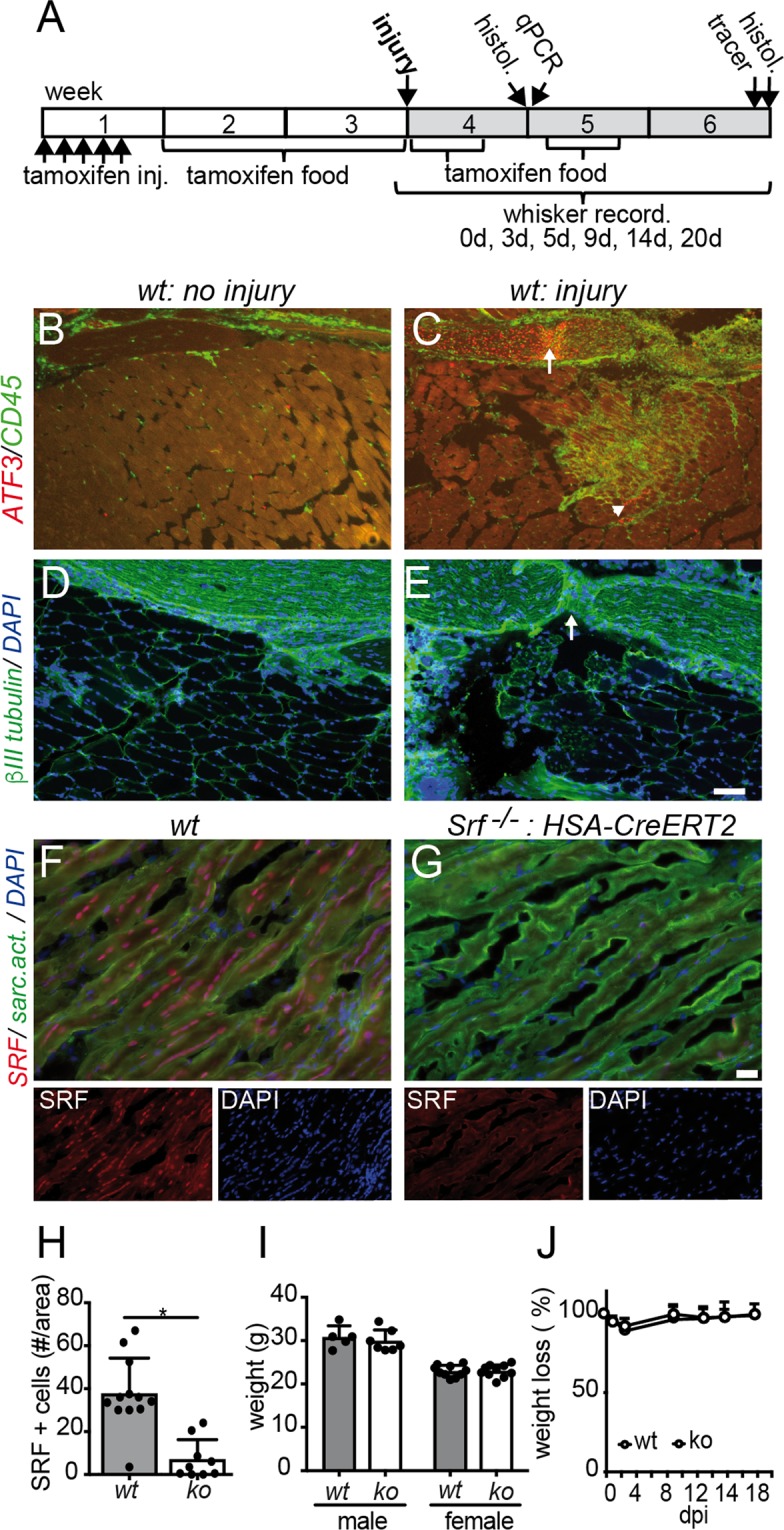
Muscle specific SRF deletion in a mouse nerve-muscle injury model (**A**) Experimental outline of tamoxifen mediated *Srf* recombination and post injury time-points for histology (“histol.”), qPCR, whisker recording and tracer injection. (**B–E**) Depiction of the nerve-muscle injury model in wt mice. After four days of injury to the nerve and masseter muscle, ATF3 was upregulated in both the severed nerve (arrow in **C**) and masseter muscle (arrowhead in **C**). Furthermore, peripheral CD45 positive macrophages were infiltrating the lesion side (**C**), in contrast to the uninjured control side of the animal (**B**). After injury, a lesion site was visible in the injured nerve (arrow in E; stained for βIII tubulin) not present on the uninjured control side (**D**). (**F–H**) In wt mice (**F**), SRF (red) was present in nuclei (labeled with DAPI in blue) of masseter myofibers (labeled with sarcomeric actinin, green) whereas SRF levels were strongly decreased in (**G**; quantified in **H**). Smaller pictures show individual SRF and DAPI channels. (**I**,**J**) SRF loss in myofibers in adult mice did not change overall body-weight neither before (**I**) nor over 20 days post injury (dpi). Each black circle in a bar reflects one animal analyzed. Data were provided as mean ± SD. *P < 0.05. Scale-bar (**B–E**) = 100 μm; (**F**,**G**) = 30 μm.

In this study we analyzed whether interference with muscle function by SRF depletion modulates the outcome of peripheral nerve regeneration. For this we employed myofiber restricted SRF ablation where Cre recombinase expression is driven by the myofiber specific human skeletal actin (HSA) promotor (*Srf*^*loxp/loxp*^*: HSA-CreERT2* mice). Peripheral nerve injury was induced at the facial nerve (FN) of adult mice whose trajectory follows several muscles of the face (see Fig. 1). The FN connects facial motorneurons (FMNs) localized in the brainstem with several target muscles including those of the whisker pad and the orbicularis oris muscle involved in lip movements^20^.

In previous studies, a stimulatory function of neuronal SRF in facial nerve regeneration and prevention of FMN degeneration was demonstrated^21,22^. Now, in this study, we show a stimulatory role of myofiber localized SRF in FN regeneration on the histological and functional level. Mechanistically, muscle restricted SRF depletion interfered with injury induced SC proliferation.

## Results

In this study SRF ablation was induced in post-mitotic myocytes due to tamoxifen mediated Cre recombinase activation in adult mice (Fig. 1A). Three weeks after first tamoxifen injection, traumatic injury to FN branches and the associated masseter muscle was performed. Subsequently, axon regeneration was analyzed at two timepoints histologically (7 and 21 days post injury, dpi) and functionally by measuring recovery of whisker movement at several timepoints post injury (Fig. 1A).

The drop tower mediated injury model employed was previously described by us (Fig. 1C,E and^23^). In the model used, the buccal and marginal FN branches were axotomized but also a cut into the underlying masseter muscle was induced. This simultaneous injury to the nerve-muscle unit resulted in induction of the regeneration-associated gene (RAG) ATF3 in the deafferented nerve (arrow in Fig. 1C) as well as the muscle (arrowhead in Fig. 1C). Furthermore, infiltration of the injury side by CD45 positive peripheral macrophages was observed (Fig. 1C). In contrast, on the contralateral un-injured side of the animal those responses were not visible (Fig. 1B). Labeling axons with βIII tubulin revealed the position of the nerve injury (Fig. 1E) not visible in the nerve at in the intact control side (Fig. 1D).

Next, we analyzed the efficacy of SRF ablation in the masseter muscle (Fig. 1F–H). SRF was localized in myofiber nuclei in wt mice (Fig. 1F) whereas strong SRF downregulation was observed in *Srf* mutant mice (*Srf* ^*loxp/loxp*^*: HSA-CreERT2* mice, “ko”; Fig. 1G; quantified in H). We did not observe any body weight reduction upon adult myofiber restricted SRF deletion without injury (Fig. 1I), in line with a previous report^15^. Furthermore, also no differential weight loss between genotypes was observed over a period of three weeks after injury (Fig. 1J).

### Motoneuron injury responses were unaltered upon SRF loss in myofibers

The FN motoneurons reside in two brainstem nuclei, one in either brain hemisphere. We applied unilateral FN injury, therefore FN cell body reactions of the injured and intact FN nucleus could be analyzed on the same section of one animal.

**Figure 2:**
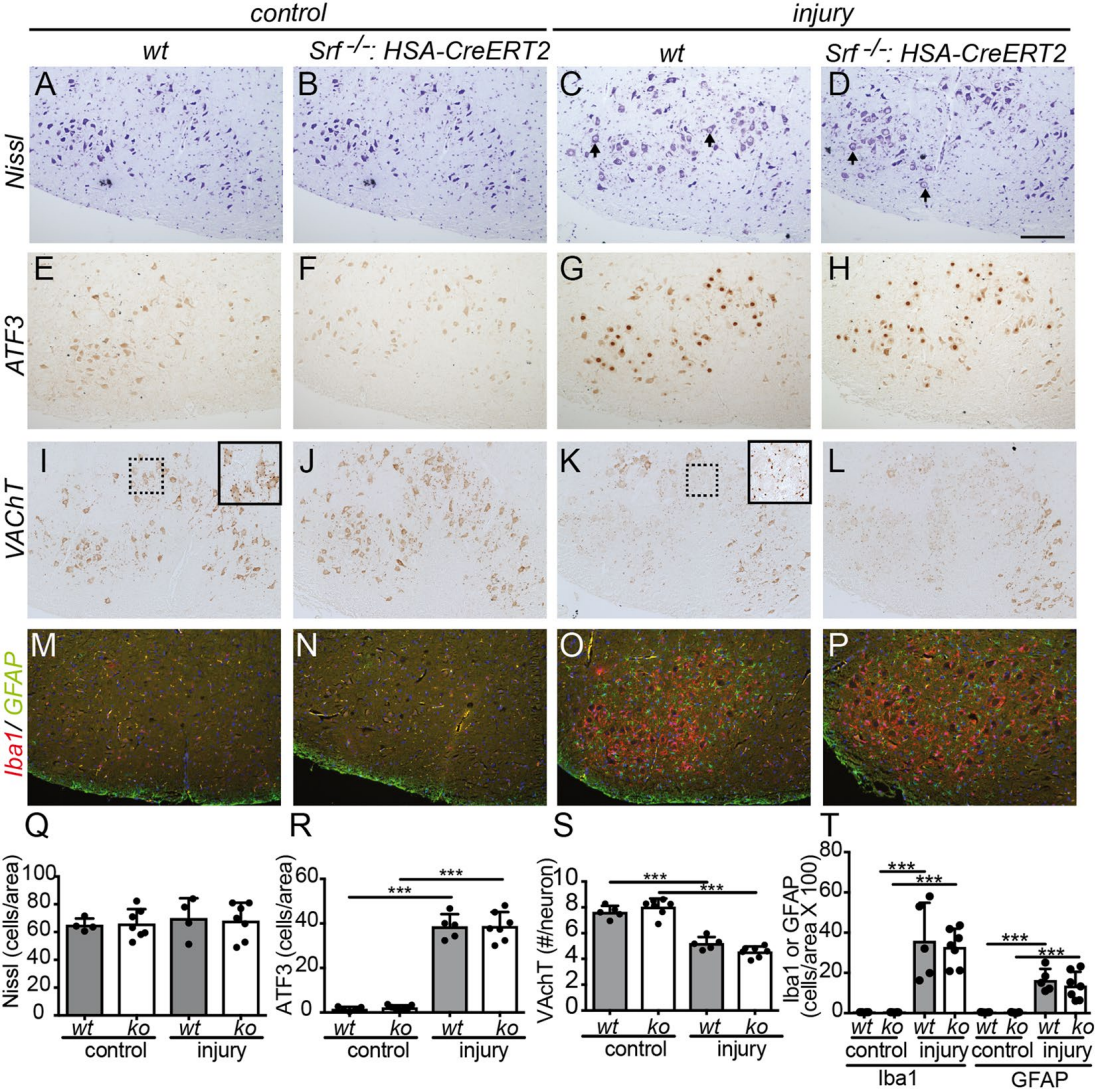
Muscle specific SRF deletion did not alter the injury response in the FN nucleus. (**A–D**) All FMNs in the brainstem FMN nucleus on uninjured (“control”; **A**,**B**) and injured (**C**,**D**) side were stained with Nissl. In the absence of injury, FMN numbers were identical between wt (**A**) and *Srf* mutant (**B**) animals (see quantification in **Q**). At 7dpi, FMNs showed typical morphological alterations (arrows in **C**,**D**). However, no differences between wt and ko, also in FMN number, were observed. (**E**–**H**) ATF3 was comparably induced in the FMN nuclei of wt (**G**) and SRF deficient (**H**) animals after injury. Without injury, ATF3 levels were strongly reduced (**E**,**F**). (**I–L**) Without injury, FMN cell bodies were decorated with VAChT positive axon terminals (**I**,**J**; see higher magnification in **I**). After injury, the VAChT signal decreased and fewer VAChT terminals were found at cell bodies (**K**,**L**; see higher magnification in **K**). No differences between wt and ko were discernible (see quantification in **S**). (**M–P**) FN injury activated the number of Iba1 positive microglial cells and GFAP positive astrocytes comparably in the FMN nucleus of wt (**O**) and myofiber SRF depleted (**P**) animals. (**Q–T**) Quantification of histological results from Nissl (**Q**), ATF3 (**R**), VAChT (**S**) and Iba1/GFAP (**T**) staining at 7 dpi. Each black circle in a bar reflects one animal analyzed. Data were provided as mean ± SD. ***P < 0.001. Scale-bar (**A–P**) = 200 **μ**m.

First of all, we analyzed a potential difference in FMN degeneration 7 dpi by labeling all motoneurons with Nissl (Fig. 2A–D,Q). However, no differences were discernable, indicating no obvious impact on myofiber SRF loss on neuronal survival post injury. ATF3 induction in neurons is a hallmark of a RAG response observed in many neuronal cell types after injury^24,25^. ATF3 induction was observed to a comparable extent in injured wt and SRF deficient animals (Fig. 2E–H,R). A further cellular response after FN injury is removal of VAChT (vesicular acetylcholine transporter) positive presynaptic terminals at motoneurons, a process termed “synaptic stripping”^26^. Indeed, 7 dpi the number of VAChT positive signals was reduced in both wt and ko animals to a similar extent (Fig. 2I–L,S). Finally, FN injury triggers a neuroinflammatory response in the deafferented FN nucleus resulting in activation of GFAP positive astrocytes and Iba1 positive microglia (Fig. 2M–P,T). Once again, no impact of myofiber restricted SRF ablation was observed and neuroinflammation was indistinguishable between genotypes.

In summary, SRF ablation in muscles has no overt impact on injury responses occurring in the FN motoneuron localized several millimeters apart from the muscle-nerve injury side.

### Facial nerve regeneration was reduced upon muscle restricted SRF depletion

**Figure 3:**
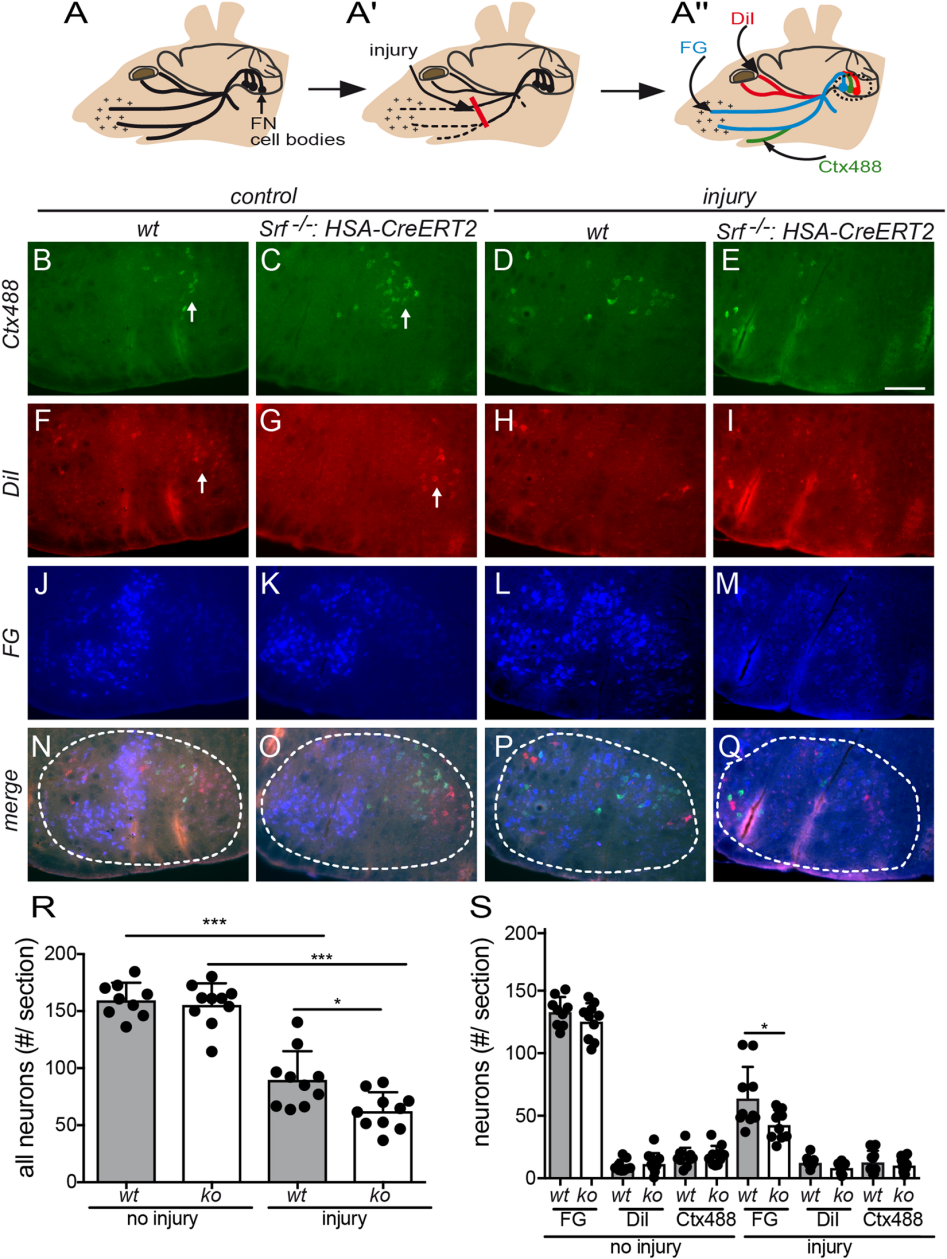
FN regeneration was reduced after SRF depletion from myofibers (**A**–A”) The FN branches connect FMN cell bodies located in the brainstem with several facial muscles, e.g. of the eyelid, lip and whisker pad (**A**). After injury of two FN branches (red bar in A’), distal FN branches degenerate. Upon successful FN regeneration, tracer molecules (FG, DiI, Ctx488) injected into different target muscles can be retrogradely transported to the FMN cell bodies. (**B–Q**) In the absence of injury, FMNs innervating the lip (labeled with Ctx488) were localized to a lateral domain of the FMN nucleus (arrows in **B**,**C**). DiI positive FMNs connected to the eyelid were also occupying a lateral position in the FMN nucleus (arrows in **F**,**G**). FG positive FMNs, representing the major FMN subpopulation, connected to the whisker pad were localized in one half of the FMN nucleus (**J**,**K**; see also merged picture in **N**,**O**). 21 dpi, the total number of labeled FMNs was reduced in wt (**D**,**H**,**L**,**P**) but more pronounced in myofiber restricted SRF deficient (**E**,**I**,**M**,**Q**) animals. Note the random localization of FMNs after injury in wt (**P**) and ko (**Q**) animals which was due to aberrant axonal sprouting. (**R**,**S**) Without injury, the number of all FMNs (irrespective of color) was identical between wt and *Srf* mutant animals (**R**). At 21 dpi, approximately 60% of wt FMNs have regenerated whereas only 40% of FMNs regenerated in SRF ablated animals (**R**). In (**S**) data were depicted for the three FMN subpopulations. For SRF deficient animals, labeled FMN neurons were reduced for all three tracers compared to wt especially for FG positive FMNs (**S**). Each black circle in a bar reflects one animal analyzed. Data were provided as mean ± SD. *P < 0.05, ***P < 0.001. White dashed lines in (**N–Q**) depict the borders of the FMN nucleus. Scale-bar (**B–Q**) = 200 μm.

Next, we analyzed the reactions of the axotomized nerves at 21 dpi. This was done by quantifying the transport efficacy of fluorescently labelled retrograde tracer molecules along the injured axons (Fig. 3^23,24^). Since the various FN branches target different muscle groups in the face, FN motoneurons in the brainstem nuclei are organized in a topographic map. Therefore, FMNs innervating the same muscle targets, e.g. whisker pad, eye or lip are grouped in subdomains in the FMN nucleus (see Fig. 3N). In order to visualize those subpopulations, three fluorescent tracers conjugated with different fluorophores (DiI, FG, Ctx488) were injected into three target areas innervated by the FN branches (see Fig. 3A). In the intact FN, those fluorescently labeled tracer molecules were efficiently retrogradely transported from the nerve terminals along the FN axons back to the motoneuron cell bodies, where numbers were quantified (Fig. 3N,O). Immediately after injury, this transport is impaired since the axonal transport route is compromised by the lesion^23,24^. However, at 21 dpi some axons have already regenerated and navigated towards new target areas which reconstitutes the transport way for tracer molecules and gives a quantitative read-out for axon regeneration (Fig. 3P–S).

In uninjured control animals of either genotype, the FMNs in the brainstem nuclei were localized according to their topographic fate (Fig. 3B,C,F,G,J,K,N,O). Thus, Ctx488 and DiI positive motoneurons innervating the lower jaw and eyelid, respectively were grouped in lateral FN nucleus positions (arrows, Fig. 3B,C,F,G). FG positive FMNs, representing the majority of motoneurons connecting to the whisker pad, occupied approximately half of the FN nucleus area in wt (Fig. 3J,N) or *Srf* mutant (Fig. 3K,O) animals.

After injury, the total number of tracer positive FMNs has declined and it was noted that in SRF deficient animals (Fig. 3Q) the numbers were even lower compared to wt animals (Fig. 3P). Quantification revealed that at 21 dpi approximately 60% of all FN axons in wt animals were tracer positive, whereas only around 40% of FMNs in SRF deficient animals incorporated one of the tracer molecules (Fig. 3R). When separately quantifying the three tracer positive FMN subpopulations, we observed that the main difference was due to altered regeneration of the FG positive FMNs, accounting for the vast majority (i.e. 65%) of all FMNs (Fig. 3S). Please note that axons of regenerating FMNs initially target topographically incorrect muscle targets due to aberrant axonal sprouting (^20^; Fig. 3P). Therefore, the topographic map in the FMN nucleus is initially distorted since these aberrant axons take up tracer molecules from topographically incorrect muscles and transport them back to the cell bodies (Fig. 3P).

In summary, muscle restricted deletion of SRF negatively affected the regeneration outcome of the facial nerve using the masseter muscle as growth surface.

### Neuromuscular junction morphology was altered by SRF depletion in muscles after injury

**Figure 4:**
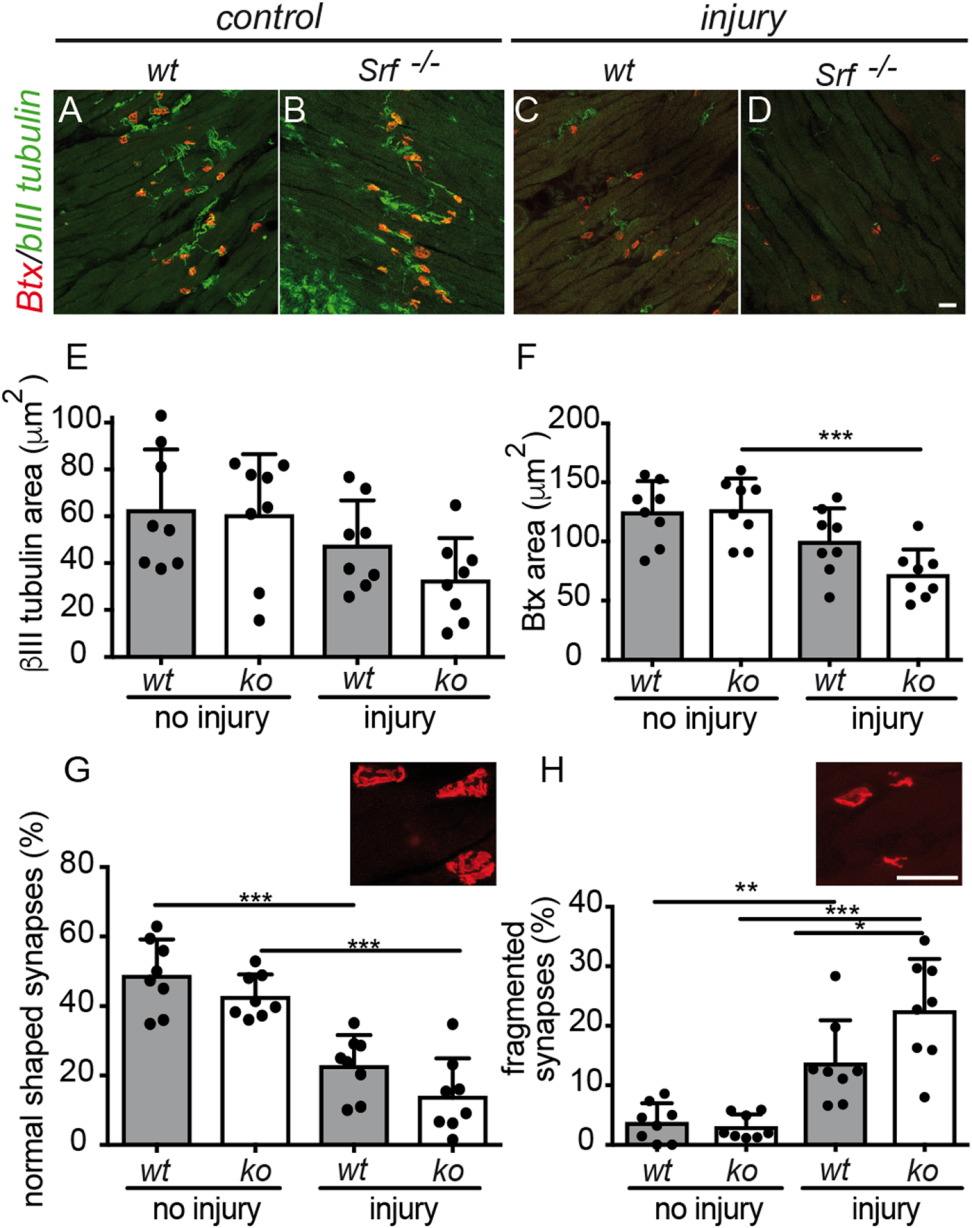
Myofiber SRF deletion induced altered NMJ morphology after injury (**A–D**) NMJs in the lip orbicularis oris muscle were stained for axon terminals with neuron specific βIII tubulin (green) and for muscular AChR with Btx (red) at 21 dpi and recorded by confocal microscopy. Without injury, many axons terminated in well elaborated NMJ structures irrespective of genotype (**A**,**B**). After injury, NMJs disintegrated and fewer axons were found in Btx positive clusters that revealed a fragmented shape (**C**,**D**). This phenotype was more prominent in SRF deficient (**D**) compared to wt (**C**) animals (see quantification in **E–H**). (**E–H**) The βIII tubulin (**E**) and Btx (**F**) area of NMJs was reduced after FN injury in wt and more pronounced in SRF deficient animals. NMJ morphologies were categorized into normally shaped (**G**) or fragmented (**H**; see example pictures). The number of normally shaped NMJs decreased after injury in wt and more so in SRF deficient animals (**G**). Conversely, fragmented NMJ abundance increased after injury in wt and more strongly in SRF ablated animals (**H**). Each black circle in a bar reflects one animal analyzed. Data were provided as mean ± SD. *P < 0.05, **P < 0.005, ***P < 0.001. Scale-bar (**A–D**; **G**,**H**) = 30 μm.

FN axons terminate at several facial muscles where they form neuromuscular junctions (NMJ^20^). In order to visualize alterations in NMJ morphology, we stained NMJs in the lip muscles (m. orbicularis oris) at 21 dpi (Fig. 4). Presynaptic axon terminals were stained with neuron-specific βIII tubulin and muscular acetylcholine receptor (AChR) clusters were labeled with α-Bungarotoxin-tetramethylrhodamine (Btx).

In the absence of axonal injury, FN axons terminated in NMJs with typical synaptic architecture showing elaborated endplates with several junctional folds (Fig. 4A,B,G). No obvious impact of myofiber SRF ablation was observed on NMJ morphology before injury (Fig. 4A,B; quantified in E-H). After injury we noted a reduction in the βIII tubulin positive axonal area and Btx positive area in the NMJs (Fig. 4C–F). This reduction was more pronounced, although statistically not significant, in SRF deficient animals (Fig. 4E,F). Furthermore, we categorized NMJs according to morphology in normal shaped and fragmented NMJs (see example pictures in Fig. 4G,H). After injury, approx. 23% of all NMJs were normally shaped in wt mice whereas in SRF deficient mice only 14% has such regular shape (Fig. 4G). When inspecting fragmented NMJs, 14% of all NMJs were fragmented after FN injury whereas this was significantly increased to almost 23% after SRF removal from muscle cells (Fig. 4H).

This finding suggests that SRF ablation in myofibers alters synaptic morphology by enhanced NMJ fragmentation after peripheral nerve injury.

### Recovery of whisker movement after injury was reduced after SRF ablation in muscles

Mice receive sensory input via whiskers whose movement is triggered by underlying muscles innervated by the FN^20^. Unilateral FN injury silences such whisker movement immediately after axotomy. In subsequent days and weeks, recovery of whisker movement can be monitored by high speed camera recording and is used as a read-out for successful functional axon regeneration^23^.

**Figure 5:**
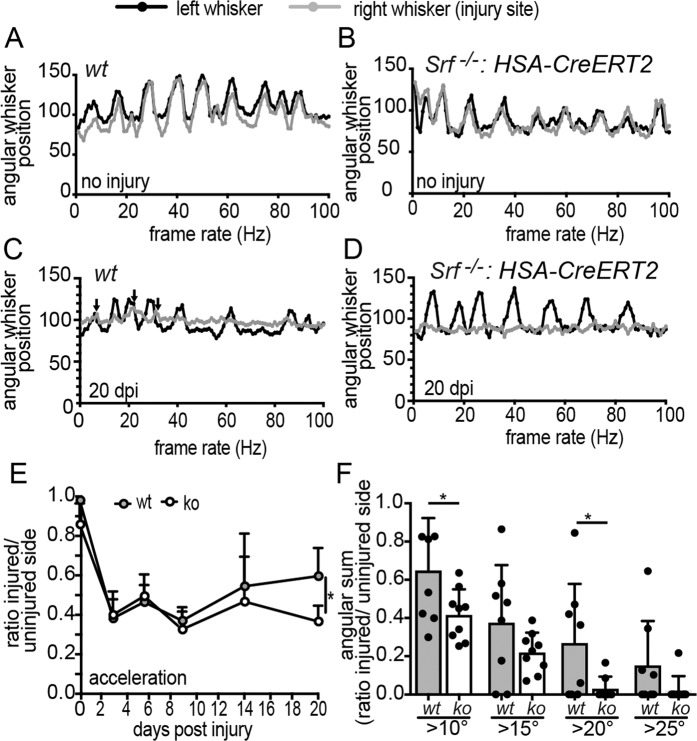
Functional recovery of whisker movement after FN injury was reduced after SRF loss in muscles. (**A–D**) Representative whisker traces of wt (**A**,**C**) and *Srf* mutant (**B**,**D**) animals before (**A**,**B**) and 20 dpi (**C**,**D**). The black lines depict whisker movement of the contralateral face, which was uninjured. The grey line depicts the ipsilateral whisker movement either before (**A**,**B**) or after injury (**C**,**D**). Before injury, both whiskers oscillated in a synchronized pattern along the 100 Hz sequence with no difference between genotypes (**A**,**B**). At 20 dpi, the whisker movement was still strongly compromised (grey lines). Nevertheless, small whisker movements with angular deflections above 10 degrees were visible in wt (arrows in **C**) but not in SRF deficient (**D**) animals (see quantification in **E**,**F**). (**E**,**F**) The angular acceleration of whisker movement was calculated as ratio between injured/uninjured side before and at five timepoints after injury. One week after injury, the ratio was decreased in wt and ko animals. At 14 dpi and statistically significant at 20 dpi, wt animals had an elevated whisker acceleration compared to ko animals (**E**). In (**F**), the angular sum of all whisker movements in a 100 Hz sequence over a certain threshold angle (10, 15, 20, 25 degrees) was depicted as ratio between injured and uninjured side. At all thresholds, wt animals had more whisker oscillations compared to SRF deficient animals (**F**). Each black circle in a bar reflects one animal analyzed. Data were provided as mean ± SD. *P < 0.05.

In order to compare the outcome of whisker movement after FN injury between wt and SRF deficient mice, animals were videotaped one day before and at five timepoints over three weeks after injury (see Fig. 1A and 5). Before nerve injury, whisker pro- and retraction is typically highly synchronous between the two whisker pads. Indeed, as seen before^23^, 100 Hz sequences of whisker movement was almost identical between both sides (Fig. 5A,B). We also observed no obvious differences between genotypes in whisker movement before injury (Fig. 5A,B,E,F).

After approximately three weeks of injury, some recovery of whisker movement was observed at the injured side of wt mice (arrows at grey line in Fig. 5C). The uninjured whisker pad was not affected and showed the typical rhythmic pattern (black line, Fig. 5C). In *Srf* ^*loxp/loxp*^*: HSA-CreERT2* mice such first small amplitudes of whisker movement at the injured side were diminished (Fig. 5D). For quantification, we measured the parameters acceleration (Fig. 5E) and the angular sum of all amplitudes above a certain threshold (Fig. 5F). For normalization, a ratio between the injured an uninjured side was calculated. In the first week after injury, no obvious differences in whisker recovery between genotypes were observed and regeneration levels were generally quite low (approx. 30% of pre-injury performance; Fig. 5E). At 14 and statistically significant at 20 dpi, whisker movement was improved in wt but not in *Srf* mutant animals (Fig. 5E). Similarly, when summing up all whisker movements over a certain threshold angle (e.g. >10 or >20 degree) at 20 dpi, it was obvious that wt mice were able to protract and retract whiskers more strongly compared to SRF lacking animals (Fig. 5F). Obviously, the higher the threshold of the angular degree was set, the lower the number of movements was. However, at all degree subgroups wt animal always had more movements compared to SRF deficient animals (Fig. 5F).

Taken together, myofiber SRF contributes to functional recovery of severed peripheral nerves.

### Myofiber restricted SRF ablation enhanced SC number but not growth factor mRNA abundance after FN injury

**Figure 6:**
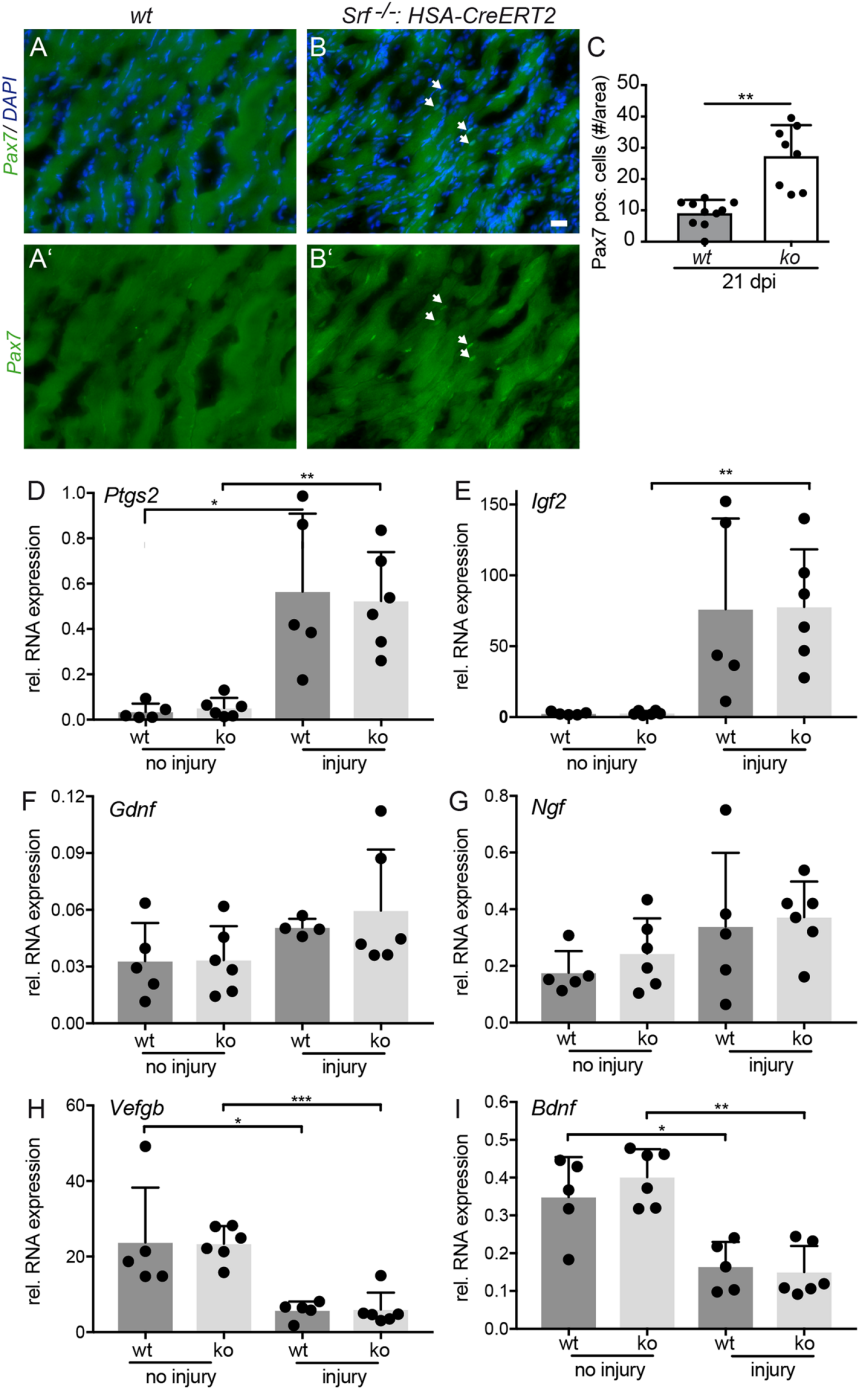
Enhanced SC numbers but no changes in growth factor mRNA levels after FN injury in SRF deficient animals. (**A–C**) Masseter muscles at 21 dpi were stained for the SC marker Pax7 (green), and DAPI (blue). Pax7 abundance was elevated in SRF depleted muscles (**B**) compared to wt myofibers. (A’,B’) show the Pax7 channel of wt (**A**) and *Srf* mutant (**B**) only. Quantification of Pax7 positive cells/area (**C**). (**D–I**) cDNA of uninjured and injured masseter muscles of wt and *Srf*^*loxp/loxp*^*: HSA-CreERT2* mutant animals (ko) was subjected to qPCR analysis for genes indicated at 7 dpi. *Ptgs2* (**D**), *Igf2* (**E**) were strongly and *Gdnf* (**F**) and *Ngf* (**G**) were mildly upregulated by injury with no differences between genotypes. *Vegfb* (**H**) and *Bdnf* (**I**) were downregulated by injury in a comparable manner between wt and ko animals. Each black circle in a bar reflects one animal analyzed. Data were provided as mean ± SD. *P < 0.05, **P < 0.001, ***P < 0.001. Scale-bar (**A**,**B**) = 30 μm.

Myofiber restricted SRF ablation was previously reported to modulate SC proliferation^12–17^. Hence, we analyzed whether this process was altered upon nerve injury in *Srf* ^*loxp/loxp*^*: HSA-CreERT2* mice (Fig. 6).

First of all, Pax7 positive SC numbers were quantified and expectedly only few Pax7 positive cells were observed per area in the intact muscle (wt: 0.85 ± 0.9 cells; *Srf* ko: 1.2 ± 1.1 cells). At 21 dpi, about ten-fold more Pax7 positive SCs were observed in the masseter muscle of wt mice (9.1 ± 1.3 cells/area; Fig. 6A) compared to approximately 27-fold more in SRF deficient muscle tissue (27.3 ± 3.5 cells/area; Fig. 6B; quantified in C). Thus, SRF ablation enhanced SC numbers after a combined nerve-muscle injury.

Finally, mRNA abundance of several growth factors (*Bdnf, Igf2, Ngf, Gdnf and Vegfb*) along with *Ptgs2* (Prostaglandin-endoperoxide synthase 2; Cox2) was quantified with quantitative real-time PCR (qPCR) in the uninjured and injured masseter muscle of wt and *Srf* mutant animals at 7 dpi (Fig. 6D–I). *Ptgs2, Igf2* and to a weaker extent *Gdnf* and *Ngf* were induced by nerve-muscle injury (Fig. 6D–G). In opposite to this, *Vegfb* and *Bdnf* were downregulated after injury (Fig. 6H,I). Comparing wt and *Srf* mutant animals, we did not observe any obvious differences between genotypes (Fig. 6D–I) suggesting no impact of myofiber restricted SRF ablation on injury-dependent modulation of growth factor abundance at this timepoint.

## Discussion

Herein we uncovered that interference with myofiber function by abrogation of the myofiber transcription factor SRF reduced peripheral nerve regeneration. Since the facial nerve analyzed in this study directly navigates along several facial muscles this suggests a paracrine effect of myofibers on injured and re-growing axons. We observed that responses in the FMN cell bodies localized in the distant brainstem nucleus and not in direct contact with myocytes were not obviously altered (Fig. 2). In contrast, regeneration processes occurring with direct nerve-muscle contact such as i) retrograde axonal tracer transport, an indirect read-out for enhanced re-growth of severed axons (Fig. 3), ii) injury dependent NMJ fragmentation (Fig. 4) and iii) whisker movement recovery (Fig. 5) relied on intact myofiber function and SRF presence. Muscles can have a beneficial function on nerve regeneration as previously noticed. For instance, early work suggested that skeletal muscle sections are well-suited substrates to bridge peripheral nerve defects^27,28^. Furthermore, coating of nerve conduits with myocytes may help to repair sciatic nerve injuries^29,30^. Thus, muscle cells may play an important role in nerve regeneration. However, a molecular basis for muscle cells’ positive influence on nerve regeneration has not been described in great detail. In this study we show that SRF, regulating a wealth of processes in myofibers and SCs might be such a novel muscle resident molecule facilitating nerve regeneration. We observed that SRF deletion in muscles affected both regeneration processes directly in the injured masseter muscle (e.g. SC proliferation; Fig. 6) but also in distant muscles such as lip muscles not directly injured (NMJ morphology; Fig. 4). This suggests that SRF in muscle exerts functions in nerve regeneration also in the denervated muscle independent of a direct muscle injury.

For neuronal SRF, such a pro-stimulatory function in axon regeneration was already demonstrated^22^. What paracrine signaling molecules might be regulated by muscular SRF affecting the growth activity of severed axons? In previous studies mRNA levels of secreted factor such as insulin-like growth factor 1 (IGF-1), IL-4 and IL-6 were altered in *Srf* mutant myofibers^16,17^. Additional growth factors, e.g. brain-derived neurotrophic factor (BDNF) and nerve growth factor (NGF) that are secreted also by the denervated muscle also stimulate neuronal regeneration^1,2^. Of note, BDNF and NGF target SRF mediated gene regulation and in turn are under SRF transcriptional control^31–33^. We analyzed mRNA levels of *Bdnf* and *Ngf* along with several other growth factors at 7 dpi but did not observe differences between wt and *Srf* mutant animals (Fig. 6). This argues against an obvious transcriptional regulation of these growth factors by SRF at least at this timepoint after injury.

Besides myofibers, SRF regulates SC function. For instance, previous literature has shown on the one hand decreased SC proliferation seven days after overload induced muscle hypertrophy in myofibers lacking SRF^16^. On the other hand, mouse mutants of MRTFs (myocardin related transcription factors), essential SRF cofactors, have shown the opposite, i.e. excessive SC proliferation^34^. However, both studies are difficult to compare since in the latter study constitutive MRTF-A deficient mice were used thereby also affecting SRF function in SCs. In any case, we likewise observed enhanced SC numbers in myofiber restricted *Srf* mutants in our muscle-nerve injury model at a later timepoint, i.e. 21 dpi (Fig. 6). Taken together, current data suggest that SC regulation by SRF can both decrease and enhance their proliferation. This might depend on muscle tissue investigated, injury type applied and also differences on the timepoint analyzed after injury. In any case previous data together with our new results (Fig. 6) point at a crucial role for SRF in SC function. So far, a role of SCs in nerve regeneration has not been reported to the best of our knowledge. However, given their important role in replacing injured myofibers, it is highly likely that SCs also contribute to the impaired nerve regeneration phenotype described in this study. Transferred to our results, high SC numbers (Fig. 6) correlated with decreased regenerative success in *Srf* mutants (Figs. 3–5) suggesting an inhibitory SC function in nerve regeneration. Interestingly, SCs secrete semaphorin family members such as Sema3A, a well-established regulator of axonal growth and regeneration^35^. Thus, future studies will have to address whether SCs influence nerve regeneration through semaphorin secretion. Furthermore, it might be interesting to specifically delete SRF from SCs (e.g. using Pax7^CreERT2^ mice^13^) to directly analyze the impact of SCs and SRF within SCs on nerve-muscle regeneration after injury.

In summary, in this study we show a role of myofibers for nerve regeneration and identified SRF as a novel muscle expressed molecule modulating this process.

## Methods

### Mice

To induce a deletion of SRF in the skeletal muscle, a mouse strain was used where tamoxifen inducible Cre recombinase expression was driven by the human α-skeletal actin (HSA) promoter (^16^; *HSA-CreERT2* line kindly provided by Dr. Athanassia Sotiropoulos, Université Paris Descartes, France). No recombination was observed outside skeletal muscle^17^. The *Srf* ^*loxp/loxp*^ strain was previously described^36,37^. At 21 days before trauma, 2 mg tamoxifen in peanut oil was daily intra-peritoneally injected for five consecutive days. In addition, tamoxifen food was fed 14 days prior trauma induction. After the trauma, animals were fed alternately with regular and tamoxifen food at one to four days post injury (dpi) and eight to 11 dpi. *Srf* mutant animals were referred to as “ko” or *Srf* ^*loxp/loxp*^*: HSA-CreERT2*, whereas wildtype (wt) animals had the genotype *Srf* ^*loxp/loxp*^ and were negative for the Cre recombinase expressing allele. All animals (wt and ko) were treated with the same tamoxifen protocol.

All experiments were in compliance with international regulations for the care and use of laboratory animals (ARRIVE guide-lines and EU Directive 2010/63/EU for animal experiments). Mouse experiments in this study were approved by the local governmental authority for animal experimentation (Regierungspräsidium Tübingen, Germany). All methods were carried out in accordance with relevant guidelines and regulations.

### Traumatic nerve injury

Traumatic facial nerve injury was performed with a drop-tower device as previously described^23^. Adult mice (14–18 weeks old) were anesthetized with sevofluorane inhalation, a skin incision was made in the area of the masseter muscle and the buccal and marginal branches of the FN were exposed. For analgesia, 0.03 mg/kg Temgesic was injected before injury. The mice were placed under the drop tower and the head was stabilized with modeling clay. Subsequently, the mice were positioned in such way that the wedge resided on top of the two FN nerve branches. To adjust penetration depth, the 3 mm spacer of the drop tower was removed. Afterwards, the weight was allowed to fall down to perform injury. Hereby, the wedge penetrates the tissue through indirect transmission of power by the falling weight. After a falling height of 104 cm, the weight hits the metal spacer. The spacer has 3 mm space to move downwards until it gets stopped through the crossbar of the tower, thereby also limiting the penetration depth of the wedge to a maximum of 3 mm. The two nerve branches were additionally cut with microscissors in case nerve stumps were not completely separated. After this, the wound was examined under a binocular and the skin was sutured. Successful traumatic facial nerve injury was documented by the absence of whisker movement. After surgery, mice were injected with 5 mg/kg of the analgesic carprofen (Rimadyl; 50 mg/mL) within the next 24 h. Since the injury was performed unilaterally, the nerve of the contralateral side remained uninjured and served as internal control.

### Fluorescent tracer injection

The application of the retrograde axonal tracers fluorogold (FG; Fluorochrome), 1,1′-Dioctadecyl-3,3,3′,3′-Tetramethylindocarbocyanine Perchlorate (DiI; Molecular Probes) and choleratoxin subunit B (Ctx488) conjugated with Alexa 488 (Ctx488; Molecular Probes) to quantify the regeneration of the facial nerve was previously described^23^. Mice were anesthetized as described above and 4 × 1 μl FG (4% in H_2_O), 2 × 1 μl of DiI (2 μg/ml in DMSO) and 2 × 1 μl of Ctx488 (1 μg/μl in PBS) were injected with a Hamilton syringe in each whisker pad, in the eyelid or lower jaw, respectively at 20 dpi. The animals were sacrificed one day after tracer injection (i.e. 21 dpi). The brains were fixed in 4% paraformaldehyde (PFA) overnight and 80 µm vibratome sections were prepared. For each animal, all tracer positive FMNs on four sections of both facial nuclei were analyzed.

### Immunostaining

Brain tissue was fixed in 4% paraformaldehyde (PFA) for one day and 5 μm paraffin sections were prepared. Masseter muscles were prepared, washed in 30% sucrose/PBS over-night at 4 degrees, embedded in OCT compound and directly frozen in liquid nitrogen-cooled isopentane. Afterwards, 10 µm cryostat sections were prepared. Prior to immunohistochemistry, sections were fixed in 4% PFA for 10 min. For immunohistochemistry, primary antibodies included anti-ATF3 (rabbit, 1:2000, # HPA001562; Atlas Antibodies), anti-Iba1 (rabbit, 1:1000, 019–19741; WAKO) anti-GFAP (mouse, 1:1000, sc-33673; Santa Cruz Biotechnology), anti-SRF (rat, 1:200; a kind gift of Prof. Dr. A. Nordheim, Tübingen University, Germany), anti-VAChT (goat, 1:1000, Merck Millipore), anti-βIII tubulin (mouse, 1:2000, Covance), anti-Pax7 (rabbit, 1:200, Thermo scientific, PA1–117), anti-sarcomeric actinin (mouse, 1:200, Abcam) and anti-CD45 (rat, 1:100, BD Pharmingen, 550539). The primary antibodies were detected by biotin conjugated secondary antibodies (1:500; Vector Laboratories) and peroxidase-based detection systems using the ABC complex (Vector Laboratories) and DAB as substrate. Alternatively, Alexa Fluor488 or 546 conjugated secondary antibodies (1:500; Molecular Probes, Life Technologies, Darmstadt, Germany) were used. α-Bungarotoxin-tetramethylrhodamine (Btx; Sigma) was diluted 1:2000 with the blocking solution and incubated on 20 μm cryostat sections with the secondary antibody for 1 h at RT. Nissl staining was performed according to standard protocol.

### Whisker movement

Whisker analysis was performed as reported before^23^. Before injury, mice were handled daily for three-five days to accustom them for videotaping. For whisker movement analysis, all whiskers except those in the C row were clipped in anesthetized mice by microscissors. Hand restraint mice were videotaped for 51 sec by a high-speed camera (Basler acA1300-60gc) at 100 Hz. Video sequences were reviewed and 1 sec fragments were further processed in Templo Software (CONTEMPLAS GmbH, Germany). The selected video sequences were analyzed by Vicon Motus 2D software (CONTEMPLAS GmbH, Germany).

The parameters acceleration of the whiskers were reported by the Vicon Motus 2D software. The parameter angular sum were calculated by a self-written MATLAB programme (developed by Hans-Georg Glöckler, Institute of Physiological Chemistry, Ulm University). Hereby, deflections of the whisker greater or equal 10°, 15°, 20° and 25° were incorporated into the calculations.

### Quantitative real-time PCR (qPCR)

Total RNA from masseter muscles was isolated with the RNeasy fibrous tissue mini kit (Qiagen, Germany) at 7 dpi. cDNA synthesis was performed with 0.75 µg RNA, random hexamers (Biomers, Ulm, Germany) and the M-MLV reverse transcriptase (Promega). RT-qPCR was performed with 2 µl of cDNA, specific primer pairs (see below) and SYBR Premix Ex Taq (Tli RNase H Plus) PCR Master Mix (TaKaRa Bio Europe, Saint-Germain-en-Laye, France) in a 10 µl reaction volume/well of a 96-well plate in a Roche Light Cycler 480 (Roche). The Ct value of a target gene was detected with the LC480 II software and the relative mRNA level of the target gene was calculated relative to the measured Ct value of the house-keeping gene *Hprt* (Hypoxanthin-Phosphoribosyl-Transferase 1). The following primer sequences were used:

*Bdnf*

Fwd: ACC ATA AGG ACG CGG ACT TG

Rev: GAG TAG AGG AGG CTC CAA AGG C

*Igf2*

Fwd: GGG AGC TTG TTG ACA CGC TT

Rev: ACG GCT TGA AGG CCT GCT

*Ptgs2*

Fwd: TGC CTC CCA CTC CAG ACT AGA

Rev: CAG CTC AGT TGA ACG CCT TTT

*Ngf*

Fwd: GGG AGC GCA TCG AGT TTT G

Rev: TAC GCT ATG CAC CTC ACT GC

*Gdnf*

Fwd: GAG AGG AAT CGG CAG GCT GCA GCT G

Rev: CAG ATA CAT CCA CAC CGT TTA GCG G

*Vegfb*

Fwd: TAG AGC TCA ACC CAG ACA CCT

Rev: GTG AAG CAG GGC CAT AAA AGC

### Microscopy, image quantification and statistical analysis

Confocal images (Fig. 4) were acquired using an LSM-700 (Carl Zeiss AG) inverted microscope, fitted with a 20x objective. Low-magnification, wide-field images were acquired with a Keyence microscope fitted with a 10x objective.

The morphological analysis of synapses was solely based on the labelling of the AChR by Btx. Hereby, the morphological appearance was defined according to the following classes: Post-synapses with a regular, so called “pretzel-like” phenotype, exhibiting several perforations, were classified as “normal”. Contrary, Btx-positive structures consisting of small, individual fragments without any perforations, were categorized as fragmented. For each animal, on average 70, but at least 40 synapses were counted. Values are indicated as percentage of synapse class (normal or fragmented) in relation to the total synapse number.

Numbers (n) of animals were indicated in figure bars or text. Statistical significance was calculated by Prism6 software with 1way ANOVA multiple comparison tests (i.e. Sidak multiple comparisons test). None of the quantification was done blind to the genotype. *, **, *** indicate p ≤ 0.05, 0.01 and 0.001, respectively. SD is provided if not mentioned otherwise.
